# Oxidative Stress and Endoplasmic Reticulum Stress Contributes to Arecoline and Its Secondary Metabolites-Induced Dyskinesia in Zebrafish Embryos

**DOI:** 10.3390/ijms24076327

**Published:** 2023-03-28

**Authors:** Wenhua Yan, Tian Zhang, Shuaiting Li, Yunpeng Wang, Li Zhu, Yu Cao, Xiaofang Lai, Huizhe Huang

**Affiliations:** 1 The Second Affiliated Hospital of Chongqing Medical University, No.76 Linjiang Road, Yuzhong District, Chongqing 400010, China; 306331@cqmu.edu.cn (W.Y.);; 2 Key Laboratory of Bio-Rheological Science and Technology, State and Local Joint Engineering Laboratory for Vascular Implants, Bioengineering College of Chongqing University, No.174 Shazhengjie, Shapingba, Chongqing 400044, China; zhangtian_728@163.com (T.Z.);; 3 Key Laboratory of Marine Biological Resources and Environment of Jiangsu Province, College of Marine Science and Fisheries, Jiangsu Ocean University, Lianyungang 222005, China

**Keywords:** arecoline, arecaidine, arecoline *N*-oxide, oxidative stress, endoplasmic reticulum stress, dyskinesia

## Abstract

Areca nut has been listed as one of the most addictive substances, along with tobacco, alcohol and caffeine. Areca nut contains seven psychoactive alkaloids; however, the effects of these alkaloids on embryonic development and motor behavior are rarely addressed in zebrafish embryo-larvae. Herein, we investigated the effects of exposure to three alkaloids (arecoline and secondary metabolites—arecaidine and arecoline *N*-oxide) on the developmental parameters, locomotive behavior, oxidative stress and transcriptome of zebrafish embryos. Zebrafish embryos exposed to different concentrations (0, 0.1, 1, 10, 100 and 1000 μM) of arecoline, arecaidine and arecoline *N*-oxide showed no changes in mortality and hatchability rates, but the malformation rate of zebrafish larvae was significantly increased in a dose-dependent manner and accompanied by changes in body length. Moreover, the swimming activity of zebrafish larvae decreased, which may be due to the increase in reactive oxygen species and the imbalance between oxidation and antioxidation. Meanwhile, transcriptome analysis showed that endoplasmic reticulum stress and the apoptosis p53 signaling pathway were significantly enriched after exposure to arecoline and arecoline *N*-oxide. However, arecaidine exposure focuses on protein synthesis and transport. These findings provide an important reference for risk assessment and early warning of areca nut alkaloid exposure.

## 1. Introduction

Areca nut abuse is widespread, with more than 600 million users worldwide. Arecoline is the fourth most commonly used psychoactive substance in the world (after alcohol, nicotine and caffeine), which further proves its importance [1]. The areca nut contains five kinds of active alkaloids; arecoline is the main component, while arecaidine, N-methylnipecotic acid, guvacine and guvacoline are secondary alkaloids [2]. At present, the majority of studies are on arecoline, which is converted into arecaidine and arecoline *N*-oxide in vivo [3]. As the main and most abundant alkaloid compound in areca nut, arecoline has been widely studied considering its mental activity and carcinogenic potential [4,5]. Arecaidine, a pyridine alkaloid, inhibits the uptake of γ-aminobutyric acid [6] or L-proline [7] by acting as a potent inhibitor. Arecoline *N*-oxide, a metabolite of arecoline, is likely an initiator of carcinogenesis and is detoxified by *N*-acetylcysteine [8]. However, despite numerous studies on these alkaloids, the mechanism behind the dyskinesia effect of areca nut compounds is not fully understood.

Arecoline and arecaidine have been detected in all or the overwhelming majority of wastewater samples from major Chinese cities (with concentrations of up to several μg/L) [9], and it is conceivable that the amount of areca alkaloid chewers must be very high. The areca alkaloids have been confirmed to be transported effectively between the mother and fetus. Arecoline has been found in the placenta and meconium of newborns born to pregnant women who chewed areca nut [10,11]. In mice, a study demonstrated that a single intraperitoneal injection of arecoline caused a lower level of locomotor activity in a dose-dependent manner [12]. In zebrafish (*Danio rerio*), a study showed that arecoline induced toxicity by reducing embryo weight and delaying embryo development [13]. In addition, another experiment showed similar results. Short-term exposure to arecoline induced growth retardation, sports injury, muscle structural changes and motor activity decline in the embryonic stage [14]. In adult zebrafish, acute exposure to both arecolines showed hyperactivity-like behaviors and tightened shoal formation. Meanwhile, social behaviors toward conspecifics and fish aggressiveness levels are affected by exposure to arecaidine [15]. These discrepancies are attractive to be reported and give us a profound explanation of how arecoline affects the animal model. Meanwhile, other compounds that are major alkaloids, such as arecaidine and arecoline *N*-oxide, are less known and less studied.

Oxidative stress and endoplasmic reticulum (ER) stress are considered contributing factors and therapeutic targets for motility dysfunction [16,17]. Accumulating evidence has demonstrated that oxidative stress overproduction mediates the toxicity of arecoline and that arecoline reduces cell activity in explant cell culture and induces hair cell apoptosis through the effect of oxidative stress, which expands the potential hazards of betel quid to common neurological disorders [18]. Arecoline may inhibit cell proliferation and promote cell apoptosis through the M2 muscarine receptor, which is directly related to an increase in intracellular ROS levels [19]. Arecoline *N*-oxide has little research on motor dysfunction, but a study demonstrated that arecoline *N*-oxide induced initial stage carcinogenesis via inflammation, ROS and depletion of antioxidant enzymes [20]. Some studies at the cell level in vitro have proven that the process of arecoline exposure causing neurotoxicity [21] or oral submucous fibrosis [22] is accompanied by ER stress. It can be speculated that the toxicity of areca alkaloids is tightly related to oxidative stress and ER stress. However, the understanding of the mechanism of areca alkaloid-induced developmental deformity and dyskinesia is still very limited.

Zebrafish (*Danio rerio*) is an ideal model for investigating alkaloid toxicity at the molecular and behavioral levels. Herein, using the zebrafish embryo model, we discovered arecoline and secondary metabolite-induced embryonic developmental disorder, a process that contributes to dyskinesia. We examined the effects of arecoline, arecaidine and arecoline *N*-oxide on embryonic development, disturbance of the oxidoreductase system, swimming activity and transcriptomic responses in zebrafish, which would provide novel insights into areca alkaloid toxicity.

## 2. Results

### 2.1. Areca Alkaloids Exposure Impair Zebrafish Early Development

**Figure 1:**
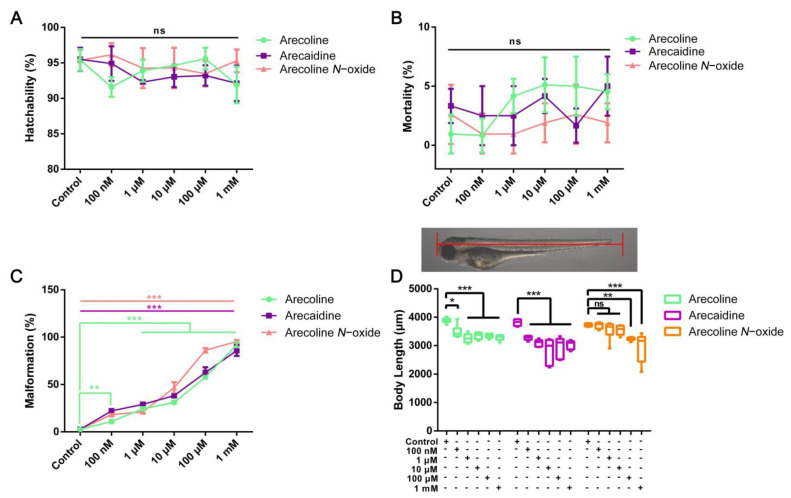
The effects of arecoline, arecaidine and arecoline *N*-oxide on embryonic development of zebrafish. (**A**) Hatchability of embryos at 72 hpf. (**B**) Mortality of zebrafish at 96 hpf. (**C**) Malformation rate of the larvae at 96 hpf. (**D**) Body length of the larvae at 96 hpf. Values are presented as the mean ± SD (*n* = 3). Asterisks denote significant differences between treatments and the control (*, *p* < 0.05; **, *p* < 0.01; ***, *p* < 0.001, ns means no significance). The mean, SD, *p* value and corresponding significance are shown in Tables S1–S4.

The potential risk of areca alkaloid exposure in a water environment was evaluated by morphological observation of zebrafish embryos. The hatchability at 72 hpf was recorded (Figure 1A, Table S1). At 72 hpf, exposure to all concentrations of arecoline, arecaidine or arecoline *N*-oxide did not significantly alter hatchability. As shown in Figure 1B, compared with the control group, there was no significant difference in the mortality rate of zebrafish embryos exposed to all concentrations of arecoline, arecaidine or arecoline *N*-oxide within 96 hpf (*p* > 0.05).

The malformation rate of zebrafish embryos exposed to arecoline, arecaidine or arecoline *N*-oxide was promoted in a dose-dependent manner compared with the control group at 96 hpf (Figure 1C, Table S3). Moreover, exposure to arecoline *N*-oxide significantly increased the rate of malformation at 100 nM, 1 μM, 10 μM, 100 μM and 1 mM, which were 18.26% (*p* < 0.001), 21.15% (*p* < 0.001), 46.67% (*p* < 0.001), 86.17% (*p* < 0.001) and 95.29% (*p* < 0.001) (vs. 2.81% in the control group), respectively. The half maximal teratogenesis concentration was defined as TC50. The TC50 of zebrafish embryos at 96 hpf was used to compare the potency of the tested compounds. The TC50 values of arecaidine and arecoline N-oxide were 116.9 µM and 16.55 µM, respectively. The data for arecoline did not comply with the calculation of TC50 and could not obtain a clear value. The data should be higher than those for arecoline N-oxide and arecoline N-oxide (Figure S4).

Meanwhile, at 96 hpf exposure to areca alkaloids, the body length of the larvae decreased in a dose-dependent manner (Figure 1D, Table S4). In comparison with the control group, the body length of zebrafish larvae at 96 hpf exposure to arecoline was decreased by 10.34% (*p* < 0.05), 16.20% (*p* < 0.001), 13.93% (*p* < 0.001), 13.98% (*p* < 0.001) and 15.55% (*p* < 0.001) at 100 nM, 1 μM, 10 μM, 100 μM and 1 mM, respectively. For arecoline, the body length of zebrafish embryos was still significantly reduced under low concentration exposure (14.12%, * *p* < 0.05 at 100 nM). However, under the stimulation of low concentrations of arecoline *N*-oxide, the body length of zebrafish embryos did not change significantly. In comparison with the control group, the body length of zebrafish larvae after 96 hpf exposure to arecoline *N*-oxide was decreased by 13.01% (*p* < 0.01) and 20.88% (*p* < 0.001) at 100 μM and 1 mM, respectively.

**Figure 2:**
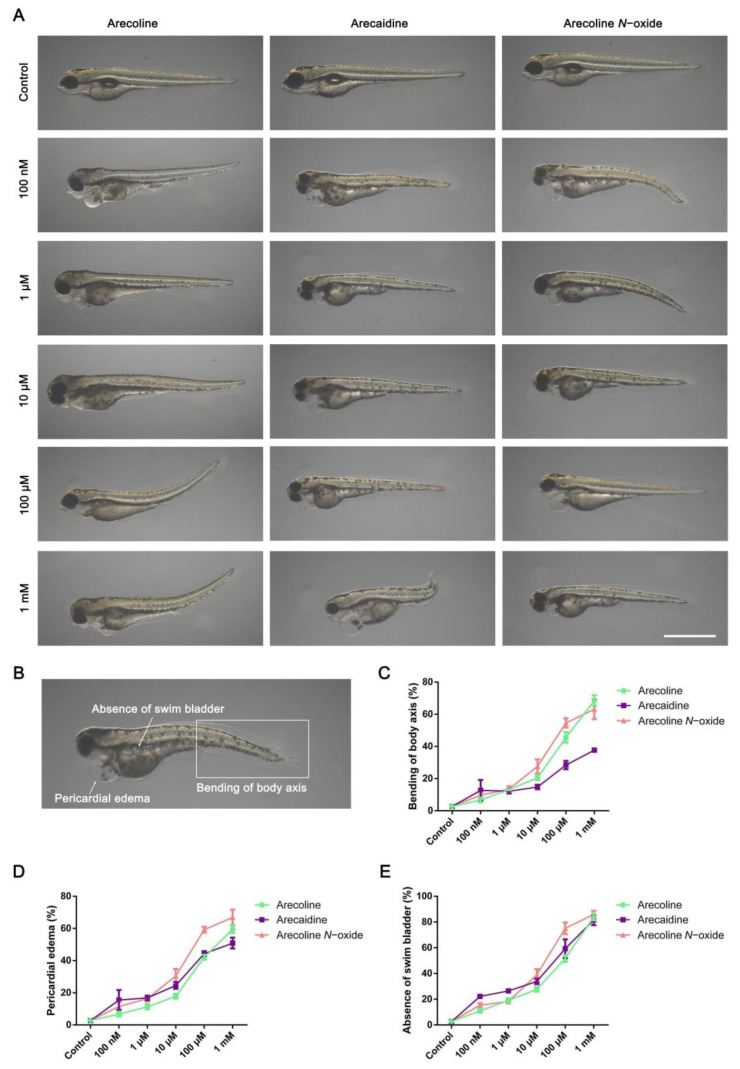
Qualitative and quantitative assay for bending of body axis, pericardial edema and absence of the swim bladder induced by arecoline, arecaidine and arecoline *N*-oxide. (**A**) Gross morphological changes after arecoline, arecaidine or arecoline *N*-oxide treatment at 96 hpf, scale bar = 1 mm. (**B**) Illustration of bending of the body axis, pericardial edema and absence of the swim bladder resulting from zebrafish embryo arecoline, arecaidine or arecoline *N*-oxide exposure. Statistics of somatic axis bending (**C**), pericardial edema (**D**) and swim bladder loss (**E**) in zebrafish embryos after exposure to arecoline, arecaidine or arecoline *N*-oxide. Values are presented as the mean ± SD (*n* = 3). The mean, SD, *p* value and corresponding significance are shown in Tables S5–S7.

Further analysis of the types of zebrafish embryonic development malformation caused by alkaloids in areca revealed that the main manifestations were body axis bending, pericardial edema and lack of a swim bladder (Figure 2A,B). In terms of body axis bending, compared with arecoline and arecoline *N*-oxide, the zebrafish embryos treated with arecaidine exhibited a lower body axis bending rate at the same concentration (Figure 2C, Table S5). For pericardial edema, zebrafish embryos treated with arecoline, arecaidine and arecoline *N*-oxide at the same concentration showed similar pericardial edema rates (Figure 2D, Table S6). Zebrafish embryos showed obvious pericardial edema under low concentration treatment, which may lead to cardiac function damage and affect blood transport. In addition, all three alkaloids in areca led to the loss of the swim bladder (Figure 2E, Table S7), which resulted in the failure of the zebrafish embryo balance and the decline of the movement ability. Taken together, these three areca alkaloid exposures impaired zebrafish early development, and the main manifestations were shortening of body length, bending of body axis, pericardial edema and loss of the swim bladder.

### 2.2. The Imbalance between Oxidation and Antioxidation Generated by Alkaloids in Zebrafish Embryos Exposed to Areca Nut Extract

**Figure 3:**
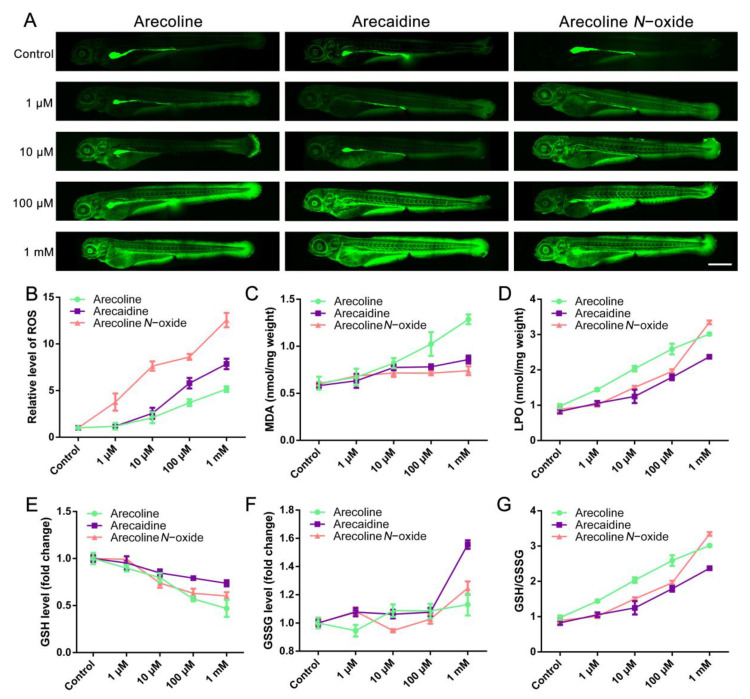
Areca alkaloids resulted in ROS, overproduction of LPO and depletion of the antioxidant molecule GSH in zebrafish embryos at 96 hpf. (**A**) ROS were observed by fluorescence microscopy, and the relative fluorescence intensity of ROS was counted. (**B**) Scale bar = 500 μm. The contents of MDA (**C**) and LPO (**D**) per mg weight were examined, as well as the fold changes in GSH (**E**), GSSG (**F**) and GSH/GSSG (**G**). Values are presented as the mean ± SD (*n* = 3). The mean, SD, *p* value and corresponding significance are shown in Tables S8–S13.

Oxidative stress is an important indicator of embryonic developmental injury. After treatment, the relative ROS level of the embryos (Figure 3A) was detected at 96 hpf. After exposure to arecoline, arecaidine or arecoline *N*-oxide, the larvae showed enhanced ROS levels with increasing exposure concentrations. The statistical results showed that even low-dose arecoline *N*-oxide-treated zebrafish embryos still had a significant increase in ROS (Figure 3B, Table S8). ROS accumulation can cause serious damage to cell structures, and ROS-induced lipid peroxidation produces MDA. We detected the MDA content in zebrafish embryos at 96 hpf and observed that arecoline, arecaidine and arecoline *N*-oxide significantly promoted the production of MDA from 10 μM. Moreover, the MDA content in the high-concentration arecoline-treated group was nearly twice that of the other two treatment groups (Figure 3C, Table S9). In addition, lipid peroxide (LPO) seemed more sensitive than MDA. Areca alkaloid exposure significantly elevated the amount of LPO in all three treated groups. Under the stimulation of high concentrations, the LPO content was more than three times that of the control group (Figure 3D, Table S10), indicating that areca alkaloids induce oxidative stress in zebrafish embryos.

Glutathione (GSH) provides the reducing equivalent and is a critical component in the cellular antioxidant system, and a tiny part is oxidized glutathione disulfide (GSSG). Here, a decrease in GSH levels was detected in alkaloids in areca-stressed embryos, which might be a stress response to the toxicity of excessive alkaloids in areca. The results showed that treatment above 10 μM arecoline, arecaidine or arecoline *N*-oxide markedly depleted GSH (Figure 3E, Table S11). Simultaneously, the increase in GSSG content was caused by excessive areca alkaloid exposure at a high dose (Figure 3F, Table S12). Finally, the proportion of GSH/GSSG more clearly shows that areca alkaloid exposure consumed GSH and accumulated GSSG, especially arecoline *N*-oxide (Figure 3G, Table S13). Together, areca alkaloid exposure impaired zebrafish early development and was relevant to the imbalance between oxidation and antioxidation.

### 2.3. Effects of Areca Alkaloids on the Motion Behaviour in Zebrafish Embryos

**Figure 4:**
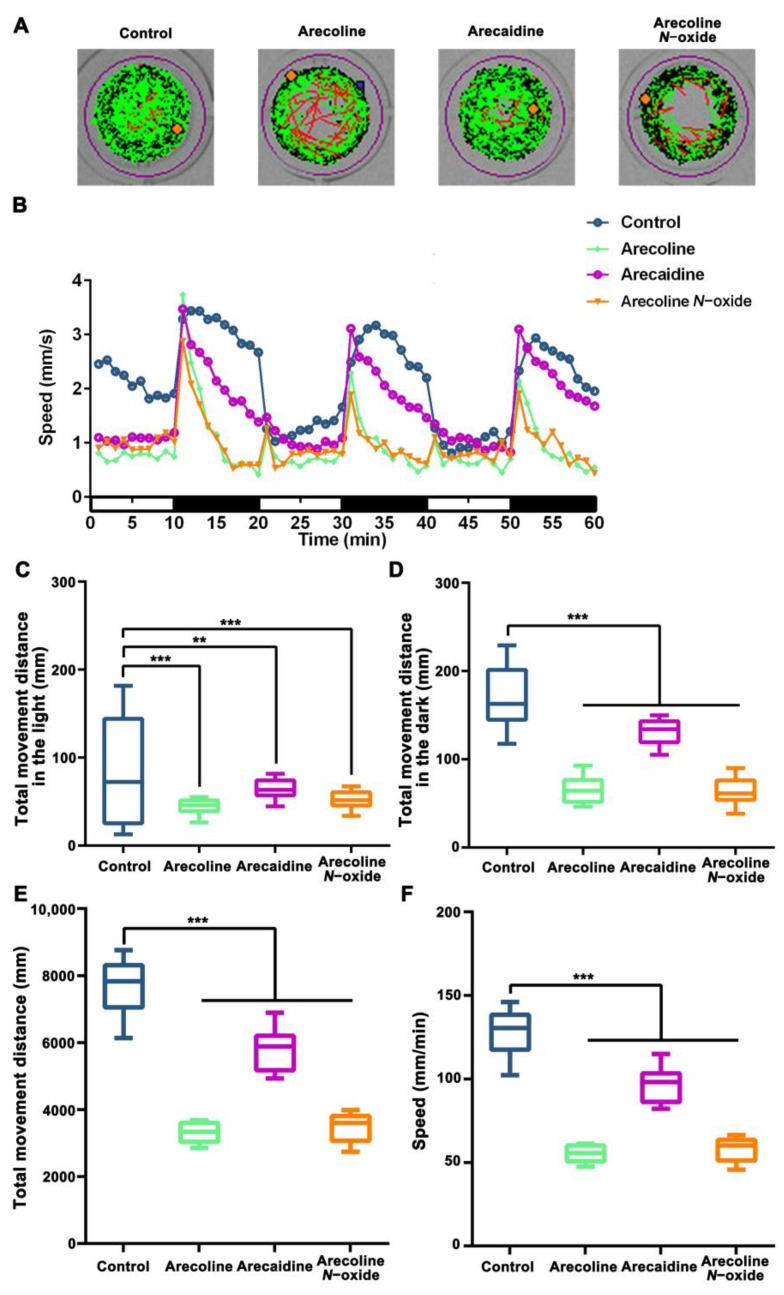
Effect of areca alkaloid exposure on the motor nervous system in zebrafish larvae at 96 hpf. (**A**) Representative trajectory traces of zebrafish larvae treated with areca alkaloids (10 μM arecoline, 10 μM arecaidine and 10 μM arecoline *N*-oxide) during 60 min light–dark stimulation tests. Black line: slow-moving distance, green line: medium-moving distance, and red line: fast-moving distance. (**B**) Swimming speeds of zebrafish larvae under light–dark stimulation tests. Total movement distance of zebrafish larvae in the light (**C**) and dark (**D**). Behavioral parameters, including total swimming distance (**E**) and velocity (**F**), of zebrafish larvae. The values are presented as the mean ± SD in histograms or as median (line), while whiskers mean min to max levels in boxplots. ** *p* < 0.01 and *** *p* < 0.001 (one-way ANOVA with uncorrected Dunn’s post-test for differences and with Tukey’s post-test for differences between treatments). The mean, SD, *p* value and corresponding significance are shown in Tables S14–S16.

The baseline locomotor performance of larvae treated with 10 μM arecoline, 10 μM arecaidine or 10 μM arecoline *N*-oxide was compared under light–dark stimulation. Under dark conditions, both the control and areca alkaloid-treated groups swam longer distances and more rapidly than during light conditions. Areca alkaloid-treated zebrafish larvae displayed worse locomotor activity than the control group in the light–dark stimulation tests (Figure 4A). Both the arecoline- and arecoline *N*-oxide-treated groups showed a similar preference for swimming in the outer zone relative to the central “open space” area of the well (Figure 4A; well illustration). At 96 hpf exposure to areca alkaloids, the larvae showed hypoactivity in both the light and dark phases (Figure 4B). The average distance traveled in the light (control = 88.81 ± 66.69, arecoline = 43.25 ± 11.43, arecaidine = 63.21 ± 14.11 and arecoline *N*-oxide = 52.31 ± 11.66; for *n* = 15 larvae; Figure 4C, Table S14) and in the dark (control = 167.2 ± 46.92, arecoline = 66.83 ± 17.76, arecaidine = 130.8 ± 16.99 and arecoline *N*-oxide = 63.25 ± 17.67; for *n* = 15 larvae; Figure 4D, Table S14) were comparable between both cohorts. Additionally, the total swimming distance in the light–dark stimulation tests traveled (control = 7679 ± 795.2, arecoline = 3302 ± 291.1, arecaidine = 5821 ± 599.8 and arecoline *N*-oxide = 3457 ± 412.6; for *n* = 15 larvae; Figure 4E, Table S15), and the average velocity (control = 128.0 ± 13.25, arecoline = 55.04 ± 4.85, arecaidine = 97.01 ± 9.99 and arecoline *N*-oxide = 57.62 ± 6.88; for *n* = 15 larvae; Figure 4F, Table S16). Therefore, early-life exposure to arecoline, arecaidine or arecoline *N*-oxide may cause significant abnormal locomotor activities in zebrafish larvae.

### 2.4. Transcriptional Responses to Areca Alkaloid Exposure

**Figure 5:**
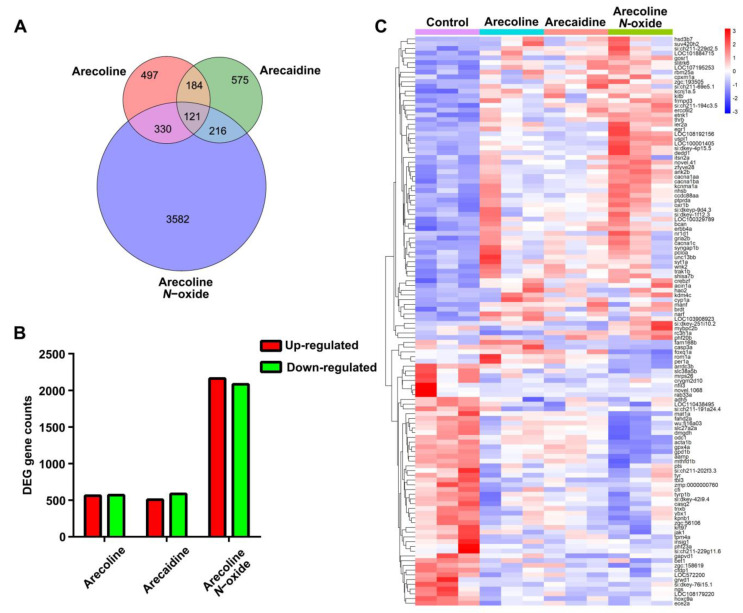
Differentially expressed genes (DEGs) in zebrafish larvae after exposure to areca alkaloids at 96 hpf. (**A**) Venn diagram of DEGs that were unique and shared. (**B**) Number of up- and downregulated genes. (**C**) Heatmap and cluster of shared DEGs.

A total of 5505 DEGs were identified in zebrafish larvae exposed to 10 μM arecoline, 10 μM arecaidine or 10 μM arecoline *N*-oxide at 96 hpf (Figure 5A). A total of 121 DEGs were shared among arecoline, arecaidine and arecoline *N*-oxide, accounting for 10.69%, 11.04%, and 2.85% of the total number, respectively. The number of upregulated DEGs was 561, 508 and 2163, while the number of downregulated DEGs was 570, 587 and 2085 in the arecoline-, arecaidine- and arecoline *N*-oxide-treated groups, respectively (Figure 5B). Cluster analysis of shared DEGs showed that the arecoline group clustered first with the arecaidine group and then formed a group with the arecoline *N*-oxide-treated group. All areca alkaloid-exposed groups clustered together, which was distinguished from the control (Figure 5C).

The GO terms of the arecoline group most represented in the categories biological process, cellular component and molecular function were oxidation-reduction process, collagen trimer and structural molecule activity, respectively (Figure S5A). The arecaidine group’s GO terms most represented in the categories biological process, cellular component and molecular function were amide biosynthetic process, nonmembrane-bound organelle and structural molecule activity, respectively (Figure S5B). Finally, the GO terms of the arecoline *N*-oxide group most represented in the categories biological process, cellular component and molecular function were nucleoside monophosphate metabolic process, nonmembrane-bound organelle and structural molecule activity, respectively (Figure S5C). All three alkaloids in the areca nut-exposed groups shared the same molecular function and structural molecule activity.

**Figure 6:**
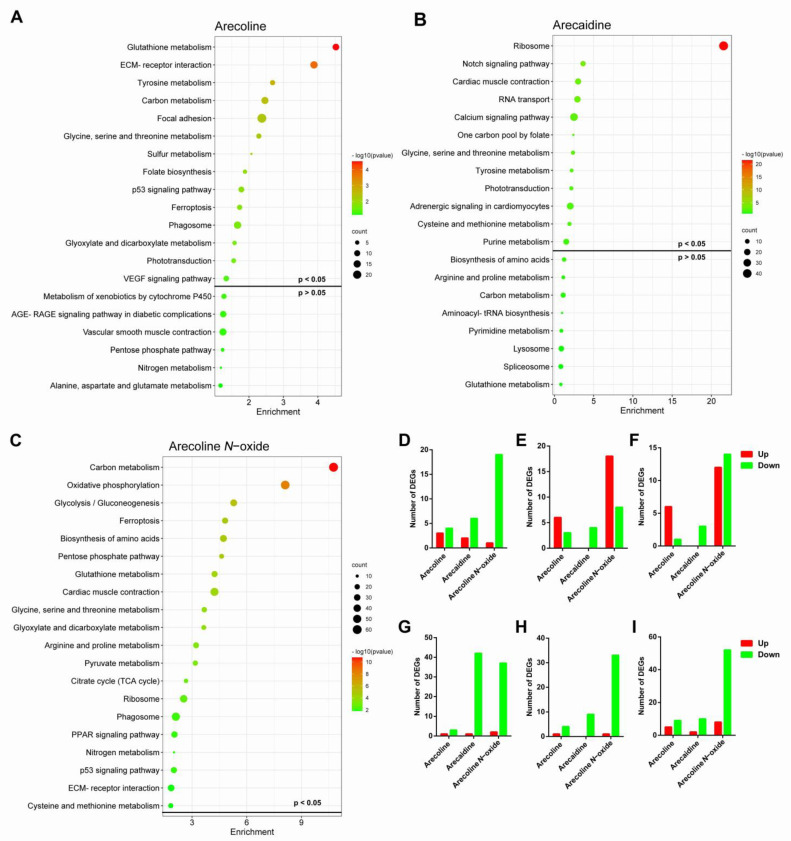
Enriched KEGG terms and the number of up- and downregulated DEGs in the different pathways in zebrafish embryos after exposure to areca alkaloids for 96 hpf. Enriched KEGG terms of arecoline (**A**), arecaidine (**B**) and arecaidine N-oxide (**C**). Number of DEGs in glycine, serine and threonine metabolism (**D**), p53 signaling pathway (**E**), ferroptosis (**F**), ribosome (**G**), biosynthesis of amino acids (**H**), and carbon metabolism (**I**).

KEGG analysis of the DEGs revealed several significantly enriched pathways (Figure 6A–C). The DEGs were significantly enriched in “glycine, serine and threonine metabolism”, where 7, 8 and 20 DEGs were confirmed after exposure to arecoline, arecaidine and arecoline *N*-oxide, respectively. Among these genes, 3, 2 and 1 genes were upregulated, while 4, 6 and 19 genes were downregulated in embryos exposed to arecoline, arecaidine and arecoline *N*-oxide, respectively (Figure 6D). The metabolic pathways involved in lipid, protein and glycogen metabolism were identified in zebrafish larvae exposed to arecoline, arecaidine and arecoline *N*-oxide (Figure 6E,F). Exposure to arecoline, arecaidine and arecoline *N*-oxide shared the same enrichment term “glycine, serine and threonine metabolism”, and exposure to arecoline and arecaidine shared “tyrosine metabolism” and “phototransduction”. The “carbon metabolism” pathway was significantly enriched only in zebrafish larvae exposed to arecoline *N*-oxide. Almost all DEGs involved in glycine, serine and ribosomes (Figure 6G), biosynthesis of amino acids (Figure 6H), and carbon metabolism (Figure 6I) were downregulated. In summary, ER stress and p53 signaling pathways may be the causes of areca alkaloid-induced malformation and death in zebrafish larvae.

**Figure 7:**
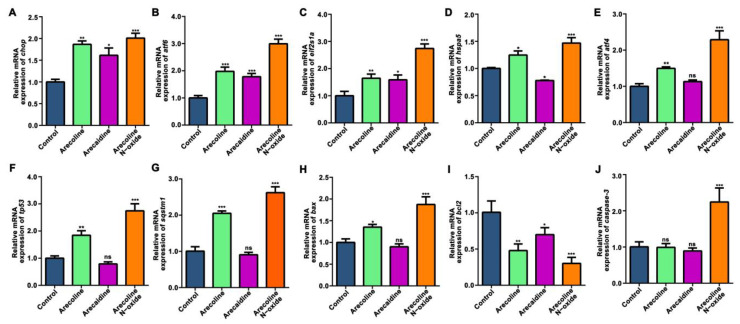
The mRNA expression levels of genes related to ER stress and the apoptosis p53 signaling pathway. The expression levels of genes related to ER stress (**A**–**E**) and the p53 signaling pathway (**F**–**J**) in zebrafish at 96 hpf. Values are presented as the mean ± SD (*n* = 3). Asterisks denote significant differences between treatments and the control (*, *p* < 0.05; **, *p* < 0.01; ***, *p* < 0.001, ns means no significance).

Exposure to arecoline *N*-oxide induced more DEGs in the ribosome, glycine, serine and threonine metabolism, and p53 signaling pathways. Many genes showed similar changes among the areca alkaloids (Figure S6). Further verified by q-PCR, exposure to arecoline, arecaidine and arecoline *N*-oxide upregulated ER stress-related genes, such as *chop*, *atf6*, *eif2s1a*, *hspa5* and *atf4* (Figure 7A–E), and changed genes involved in the p53 signaling pathway, such as *tp53*, *sqstm1*, *bax*, *bcl2* and *caspase-3* (Figure 7F–J), in zebrafish larvae at 96 hpf.

## 3. Discussion

In the present study, we demonstrated that arecoline *N*-oxide has a relatively severe toxicity of these three areca alkaloids. Arecoline *N*-oxide-treated zebrafish embryos showed more severe developmental malformations, oxidative stress levels, and relatively less locomotor behavior, induced oxidative stress, ER stress, apoptosis and metabolic disturbances with compensatory responses toward the upregulation of ribosome, protein processing in the ER, and protein export pathways by examining transcriptomic and biochemical levels, which will provide insight into fish molecular toxicological responses to areca alkaloids.

Arecoline and arecaidine were detected in all or the overwhelming majority of wastewater samples from major Chinese cities (with concentrations up to several μg/L, close to the lowest exposure concentration of our study design) [9], it is conceivable that the amount of areca alkaloid chewers must be very high. The areca alkaloids have been confirmed to be transported effectively between the mother and fetus. Arecoline was found in the placenta and meconium of newborns born to pregnant women who chewed areca nut [10,11], which leads to the influence of areca alkaloids during embryonic redevelopment. In a mouse study, arecoline decreased the number of implanted embryos and trophoblast outgrowth expansions of blastocysts in early pregnant mice [23]. In our study, arecoline, arecaidine and arecoline *N*-oxide-exposed zebrafish embryos exhibited shorter body length, pericardial edema, loss of swim bladder and body axis curvature. Previous arecoline-exposed zebrafish embryos exhibited a high incidence of pericardial edema, axial-tail curvature, and external morphological malformations [14], which was consistent with our study.

The serious developmental deformity caused by areca alkaloids will inevitably lead to the occurrence of dyskinesia. In a rodent exposure experiment, less physical activity, tremors and body curls were observed in areca semen aqueous extract-treated rats [24]. In our study, zebrafish embryos treated with these three alkaloids showed a weakening of locomotion ability, a bias toward peripheral movement and an intensification of movement after light and dark changes. The latest research found that arecoline and arecaidine induced hyperactivity-like behaviors in zebrafish larvae [15]. The exposure experiment was conducted 4 days after embryonic development, and the effect of arecoline or arecaidine exposure on development was not considered. However, the intensification of movement is consistent with the increase in the proportion of our fast movement. According to the adult zebrafish behavioral test, arecoline was found to slightly increase locomotor activity and cause robust anxiolytic-like behavioral effects in adult zebrafish [25]. Meanwhile, adult zebrafish exposed to arecaidine have reduced aggressiveness and conspecific social interaction [15].

Intriguingly, after being treated with arecoline *N*-oxide, the zebrafish larvae displayed a decrease in locomotion activity, which was similar when treated with arecoline. At present, there are few studies on arecoline *N*-oxide. They mainly focus on oral fibrosis [26] and oral squamous cell carcinoma [20,27]. The former is regulated by Caspase-8, while the latter is regulated by Notch1 and Fat1. In addition, arecoline *N*-oxide can cause liver toxicity through DNA damage and ROS [28]. The hypoactivity of zebrafish embryos by exposure to arecoline *N*-oxide may be caused by the increase in ROS and the imbalance of redox status. In addition, areca nut extract component-induced oral mucosa fibrosis contraction was related to actin filament polymerization due to ROS production [29]. One of these, arecoline, could induce neuronal apoptotic death by attenuating antioxidant defense and enhancing oxidative stress [30]. In our study, arecoline, arecaidine and arecoline *N*-oxide exposure all caused ROS overproduction in zebrafish embryos. The increase in MDA and LPO fits with the redox imbalance, while the occurrence of GSH depletion and GSSG accumulation after arecoline or arecoline *N*-oxide exposure is consistent with ferroptosis enriched in transcriptome sequencing. These results indicate that oxidative stress was activated in zebrafish embryos exposed to areca nut alkaloids and played an important role in the developmental toxicity of areca nut alkaloids.

In this study, we first carried out high-throughput sequencing to investigate the effects of arecoline arecaidine and arecoline *N*-oxide on transcriptomic changes in zebrafish embryos. Notably, GO and KEGG enrichment analyses indicated that a large number of genes were enriched in the ER stress-related pathways and the p53 signaling pathway.

There is a close relationship between oxidative stress and ER stress. ER stress can increase the content of ROS and induce oxidative stress [31], while oxidative stress can hinder the proper folding and transport of proteins, calcium homeostasis and stimulate ER stress [32]. Therefore, we detected the expression of ER stress-related genes. ER stress causes the accumulation of misfolded proteins in the ER, activating the transcription factor, atf6, which induces ER stress response genes [33]. Chemical treatment under the same conditions further heightened hspa5 levels but also eif2α/atf4 levels, both of which are known to promote antioxidant protection during ER stress [34]. Hspa5 is a zebrafish homologue of the molecular chaperone grp78 [35], which acts as a central regulator of ER homeostasis [36]. Under disturbed circumstances, grp78 binds to ire1α, perk and atf6 and is activated by the accumulation of misfolded proteins in the ER [37]. Previous reports have demonstrated that the activation of the UPR is characterized by the phosphorylation of eif2α [38]. Chop is a key mediator of ER stress and is induced when ER stress is prolonged or too severe for the organism to resolve [39]. In our study, we found that the expression levels of *chop*, *atf6*, *eif2s1a*, *hspa5* and *atf4* were significantly upregulated, which indicated that ER stress was activated in zebrafish embryos against areca nut alkaloid-induced toxicity.

Chop is an endoplasmic reticulum stress-induced apoptosis marker protein. When ER stress is activated, chop is significantly increased. The increased chop will cause an increase in bax, bcl-2 and bcl-xl, activation of caspase and cytochrome c release and induce apoptosis in related cells [40]. p53 and sqstm1 (p62) are multifunctional regulation proteins. Their activation can regulate the expression of apoptosis-inducing target genes [41,42], and bcl-2 is a type of apoptosis inhibition factor [43]. In this experiment, the exposure of areca nut alkaloids can significantly change the expression levels of tp53, sqstm1, bax, bcl2 and caspase-3. These results indicated that the apoptosis p53 pathway was induced by areca nut alkaloids in zebrafish embryos.

## 4. Materials and Methods

### 4.1. Chemicals

Arecoline hydrochloride (Cas No: 61-94-9, purity: ≥98%), arecaidine hydrochloride (Cas No: 6018-28-6, purity: ≥98%) and dimethyl sulfoxide (DMSO, Cas No: 67-68-5, purity: ≥99%) were purchased from MedChemExpress. The arecoline metabolite is not commercially available. Therefore, we synthesized the arecoline metabolite, arecoline *N*-oxide. The suspension of arecoline in ether was treated with peracetic acid at 0 °C and stirred for 1 h. After incubation, the oily base layer was separated and then precipitated five times by the addition of ether. The resulting solution was then concentrated to form arecoline *N*-oxide as a pale yellow viscous oil [20]. All synthesized chemicals were confirmed by high-resolution mass spectrometry (Supplementary Figures S1–S3). The other chemicals employed in this investigation were of analytical grade.

### 4.2. Zebrafish Maintenance and Toxicity Tests

Adult zebrafish (wild-type, AB strain) were obtained from the China Zebrafish Resource Center (Wuhan, China). The fish were reared in dechlorinated tap water at a temperature of 28 ± 0.5 °C, and the light–dark cycle was 14 h/10 h. Fertilized eggs were collected and examined under a stereomicroscope. Zebrafish embryos 6 h postfertilization (hpf) were exposed to 5 mL of arecoline, arecaidine or arecoline *N*-oxide solutions at 0.1, 1, 10, 100 and 1000 μM (nominal concentration). Fertilized eggs (n = 30 in each well) were transferred into 6-well plates with the test solutions and incubated at 26 °C under a 14:10 h day/night light regime, as seen previously. For the preliminary experiments of arecoline, arecaidine and arecoline *N*-oxide, n = 30 larvae were used for each experimental group, and the experiment was repeated 3 times [44]. The exposure solution was obtained by diluting the arecoline, arecaidine or arecoline *N*-oxide stock solutions dissolved in DMSO with embryo medium (NaCl (29.4 g/100 mL), KCl (1.27 g/100 mL), CaCl_2_•2H_2_O (4.85 g/100 mL) and MgSO_4_•7H_2_O (8.13 g/100 mL)). The final DMSO concentration in the solvent control group and all treatment groups was 0.1% (vol/vol). The exposure concentrations of 0.1 and 1000 μM were determined to be blood or salivary realistic based on previous areca nut chewing in human volunteers’ data [45,46]. The experiment was performed in triplicate. The dead embryos were removed, and the exposure solutions were refreshed every 24 h during the experiment. The morphological changes in developing zebrafish embryos were analyzed, including hatchability (at 96 hpf), malformation rate (at 96 hpf), mortality (at 96 hpf) and body length (at 96 hpf). This study was conducted in accordance with the Guidelines for the Care and Use of Laboratory Animals issued by the Second Affiliated Hospital of Chongqing Medical University.

### 4.3. Antioxidant Enzyme Analysis

The larvae at 96 hpf from each plate (n = 40 for each experimental group) were homogenized in cold sample extraction reagent (pH 7.4) and centrifuged at 8000× *g* for 10 min at 4 °C. The supernatant obtained was used to determine the levels of protein, malondialdehyde (MDA), lipid hydroperoxide (LPO), reduced glutathione (GSH) and oxidized glutathione (GSSG) using BCA Protein, MDA, LPO, GSH and GSSG Assay Kits (Solarbio, China), respectively, according to the manufacturer’s protocol. Absorbance was measured using a FlexStation 3 Multi-Mode Microplate Reader (Molecular Devices, San Jose, CA, USA).

### 4.4. Reactive Oxygen Species (ROS) Analysis

ROS fluorescence intensity was measured using 2′,7′-dichlorodihydrofluorescein diacetate (DCFH-DA), according to the manufacturer’s protocol (MCE, Junction, NJ, USA). Briefly, the larvae at 96 hpf from each plate (*n* = 15 for each experimental group) were transferred into a 24-well plate containing DCFH-DA solution (10 μM) and kept at 28 °C for 1 h in the dark. After washing and anesthesia (0.1% MS-222, Sigma-Aldrich, St. Louis, MO, USA), the zebrafish larvae were observed with a laser scanning confocal microscope (Lecia, SP8, Germany). The fluorescence intensity of individual zebrafish larvae was quantitatively analyzed using ImageJ software (NIH, Bethesda, MD, USA).

### 4.5. Larval Locomotion Tracking

Larval locomotion tracking was carried out based on previous studies [47]. Briefly, the locomotor behavior of zebrafish larvae was analyzed using the ZebraLab behavioral monitoring system (Viewpoint Life Science, Lyon, France). Larvae at 96 hpf (6 larvae per container and 18 larvae per treatment group) were randomly selected from the arecoline, arecaidine or arecoline *N*-oxide treatment groups, transferred into a 96-well plate (1 larva per well) containing dechlorinated tap water and acclimated for 30 min under light conditions. The behavioral test protocol was followed by 10 min of dark–light–dark after 10 min of acclimation of the fish in the instrument. The data were collected every 1 min.

### 4.6. Quantitative Real-Time PCR (qPCR) Assay

Total RNA from 30 larvae per replicate was isolated using an RNA isolation kit (Takara, Cat# 9108). qRT-PCR for *atf4*, *atf6*, *eif2s1a*, *hspa5*, *chop*, *tp53*, *sqstm1*, *bax*, *bcl2*, *caspase-3*, *gpx4b*, *ascl4*, *fth1*, *nox1* and *ptgs2* was performed on cDNA generated from 1000 ng of total RNA using the protocol of the qRT-PCR mRNA Detection Kit (Takara, Cat# RR047A). The gene primer sequences are shown in Supplementary Table S17. Amplification and detection of specific products were performed with a Roche Lightcycler 480 Detection System. As an internal control, β-Actin was used for the other template normalizations. Fluorescent signals were normalized to an internal reference, and the threshold cycle (Ct) was set within the exponential phase of the PCR. The relative gene expression was calculated by comparing the cycle times for each PCR target. The target PCR Ct values were normalized by subtracting the β-Actin Ct value, which generated the ΔCt value. The relative expression level between treatments was then calculated using the following equation: relative gene expression = 2 ^−(Δctsample−Δctcontrol)^.

### 4.7. RNA-Seq Analysis

Larvae at 96 hpf were collected from the 10 μM arecoline treatment group, 10 μM arecaidine treatment group or 10 μM arecoline *N*-oxide treatment group and the solvent control group for RNA extraction and RNA-Seq analysis. The quality of the RNA for sequencing was determined using a Bioptic Qsep100 Bioanalyzer. Library preparation was performed using the KAPA RNA-Seq Library Preparation Kit (KAPA Biosystems Cat# 07960140001), and the RNAs were single-end sequenced on Illumina HiSeq 3000 machines. The reads were aligned using HISAT2 with the default parameters for the rat genome. Transcript assembly and differential expression were determined using Cufflinks with Refseq mRNAs to guide assembly. RNA-seq data were analyzed using the ummerbund package in R. The heatmap was generated with a Heatmap Builder. Gene set enrichment analysis was performed using the GSEA app (Broad Institute) based on the Kyoto Encyclopedia of Genes and Genomes (KEGG) gene lists.

### 4.8. Statistical Analysis

Statistical analyses in this study were performed using GraphPad Prism 8 software. Data subjected to statistical tests were first checked for normality and homoscedasticity. If assumptions of normality and homogeneity of variance were tenable, an unpaired Student’s *t* test or ANOVA (analysis of variance), followed by multiple comparison posttest was used to determine significant differences. In cases where assumptions of normality and homogeneity of variance were not met, the Mann-Whitney test or the nonparametric Kruskal-Wallis test followed by multiple comparison test was performed. *p* values < 0.05 were considered statistically significant. Statistical differences of *p* < 0.05, *p* < 0.01 or *p* < 0.001 are represented by *, ** or ***, respectively.

## 5. Conclusion

In this zebrafish study, by using transcriptomic and behavioral analysis, in vivo ROS imaging and many biochemical assays, we revealed that exposure of developing larvae to areca alkaloids (arecoline, arecaidine or arecoline *N*-oxide) induces significant behavioral and developmental toxicity. Briefly, these alkaloid exposure resulted in imbalances in oxidative and antioxidant systems that manifested in increased ROS levels, depletion of GSH, decreased antioxidant enzyme activity in the zebrafish larvae, and induced various developmental defects involving bending of the body axis, pericardial edema and loss of the swim bladder. However, we did not find significant changes in hatchability and mortality rates after 96 hpf exposure. Transcriptome analysis of differentially expressed genes and qRT-PCR confirmed that ER stress and the p53 signaling pathway may be novel targets of areca alkaloids, especially in arecoline *N*-oxide. This study improves our understanding of the potential toxicological mechanisms of arecoline, arecaidine and arecoline *N*-oxide on aquatic organisms and will also provide valuable information for further research.

## Data Availability

Data are contained within the article.

## Supplementary Materials

The following supporting information can be downloaded at: https://www.mdpi.com/article/10.3390/ijms24076327/s1.
