# Supplementary material for: Oxidative Stress and Endoplasmic Reticulum Stress Contributes to Arecoline and Its Secondary Metabolites-Induced Dyskinesia in Zebrafish Embryos
Source: Int J Mol Sci. 2023 Mar 28;24(7):6327. doi: 10.3390/ijms24076327 (PMC10094114; doi:10.3390/ijms24076327)
Supplement: Supplementary file 1 [file ijms-24-06327-s001.zip › ijms-2270411-supplementary.pdf]

Supplementary materials:

Oxidative Stress and Endoplasmic Reticulum Stress Contributes to Arecoline and Its Secondary Metabolites-induced Dyskinesia in Zebrafish Embryo

Wenhua Yan[1†], Tian Zhang[2†], Shuaiting Li[1†], Yunpeng Wang[1], Li Zhu[2], Yu Cao[2], Xiaofang Lai[3], Huizhe Huang[1*]

1. The Second Affiliated Hospital of Chongqing Medical University, No.76 Linjiang Road, Yuzhong District, Chongqing, 400010, People's Republic of China.

2. Key Laboratory of Bio-Rheological Science and Technology, State and Local Joint Engineering Laboratory for Vascular Implants, Bioengineering College of Chongqing, University, No.174 Shazhengjie, Shapingba, Chongqing, 400044, People's Republic of China.

3. College of Marine Science and Fisheries, Key Laboratory of Marine Biological Resources and Environment of Jiangsu Province, Jiangsu Ocean University, Lianyungang, 222005, People's Republic of China.

* Correspondence: Huizhe Huang, E-mail: 102582@cqmu.edu.cn, Tel.: +86-023-62888334.

† These authors contributed equally to this work.

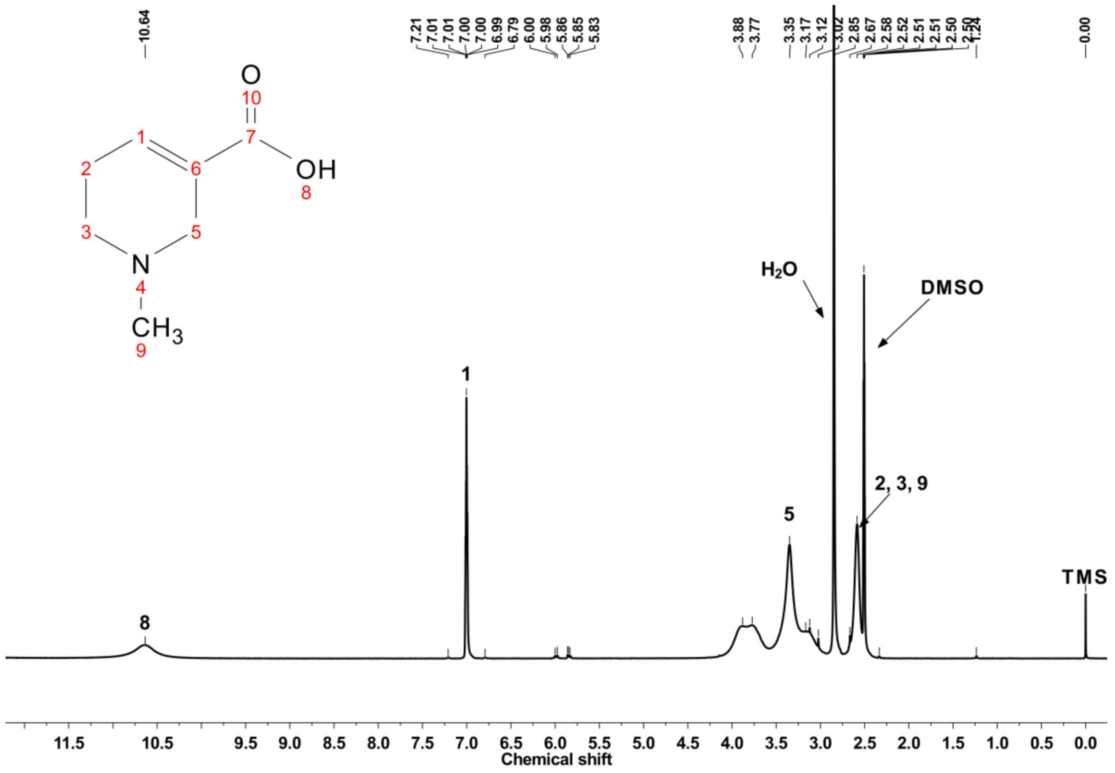

Figure S1. Nuclear magnetic resonance hydrogen spectrum (^{1}H−NMR) of arecoline (MHz, DMSO−d_6).

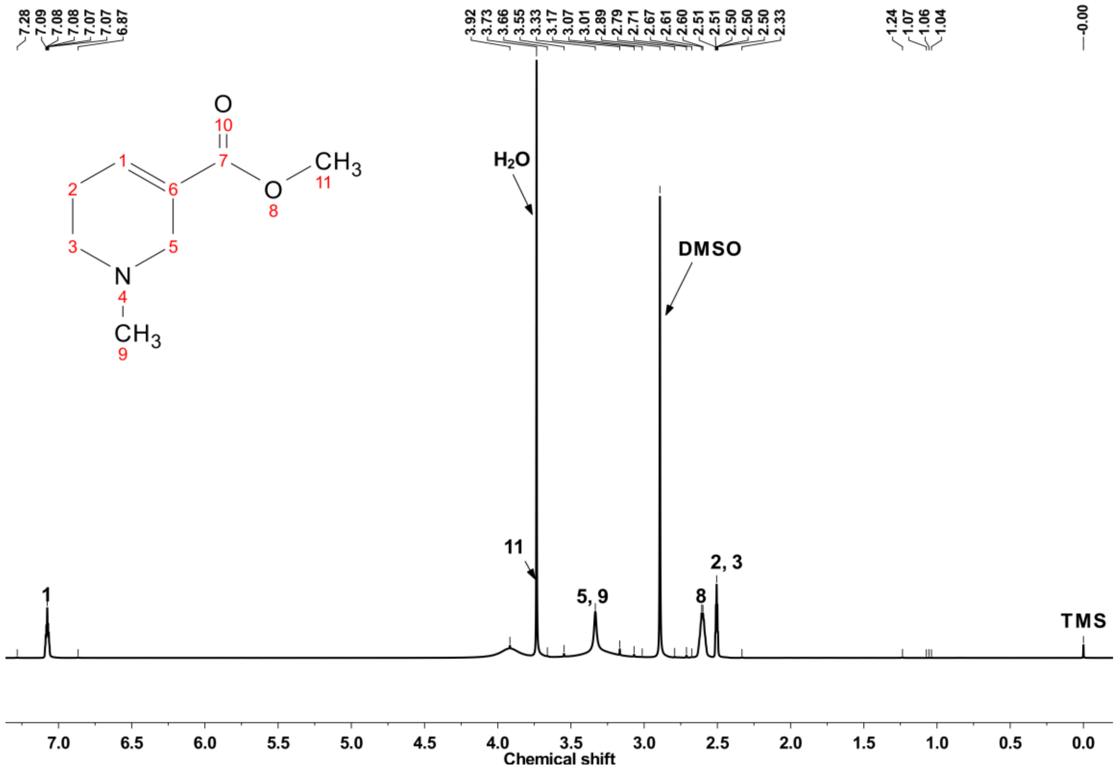

Figure S2. ^{1}H−NMR of arecaidine (MHz, DMSO−d_6).

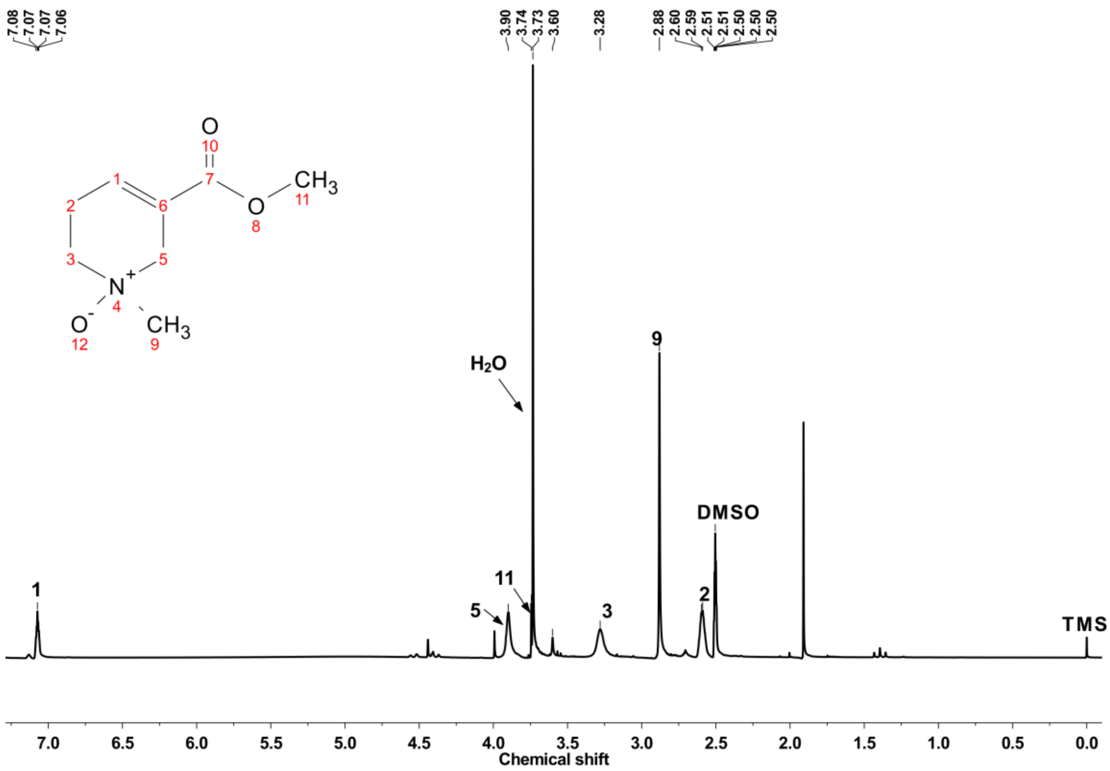

Figure S3. ^{1}H−NMR of arecoline *N*−oxide (MHz, DMSO−d_6).

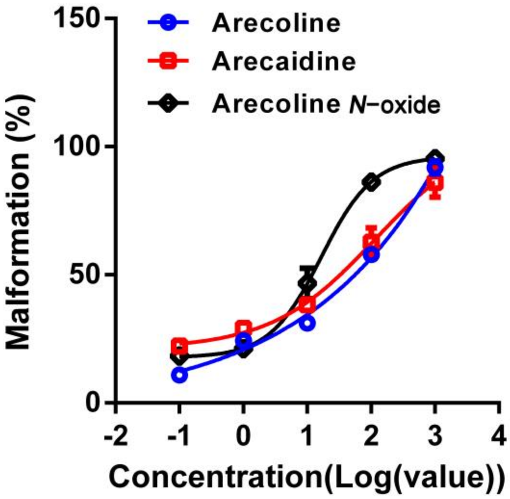

Figure S4. Half maximal teratogenesis concentration of zebrafish embryos at 96 hpf.

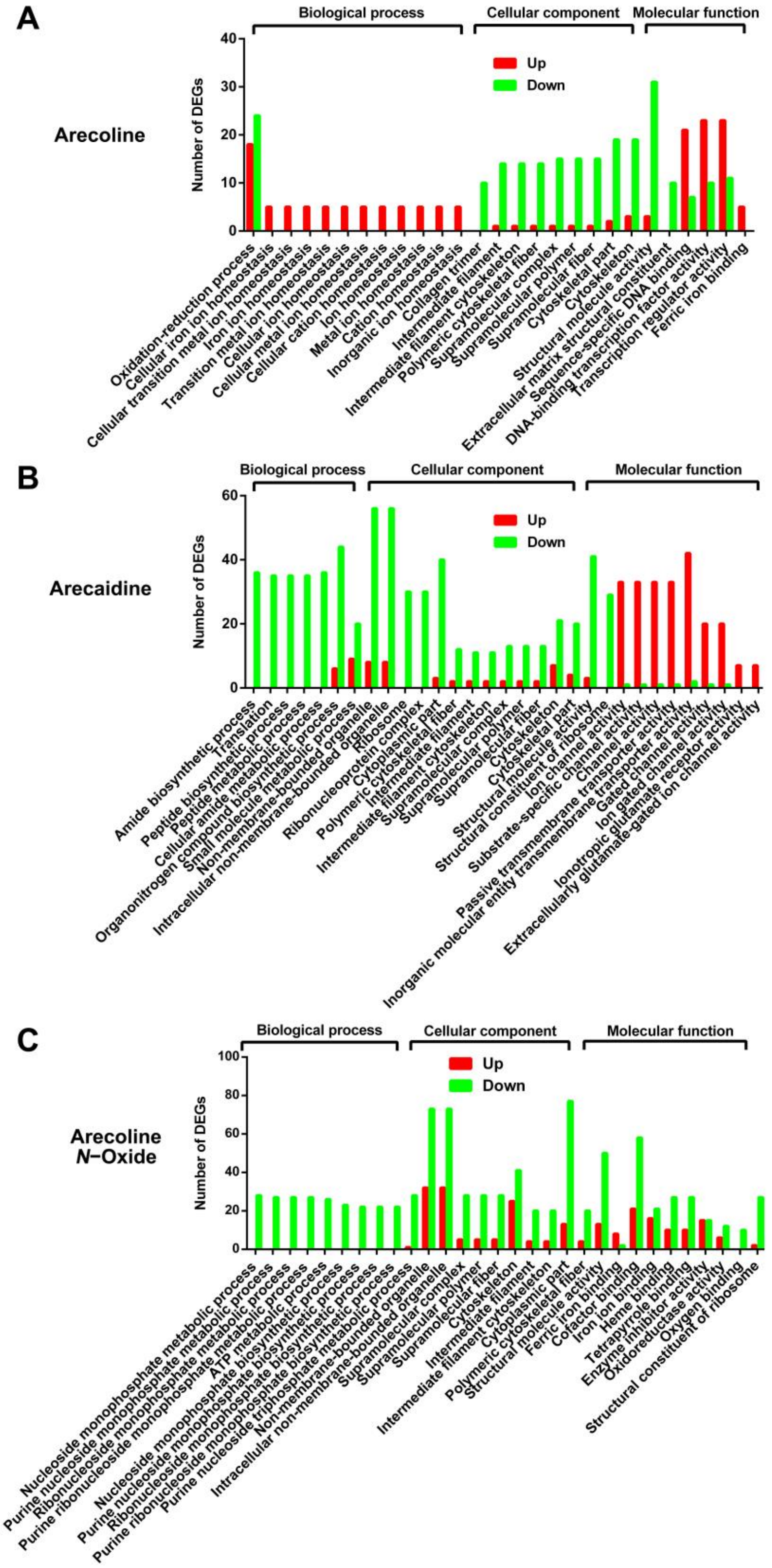

Figure S5. Genes categorized by GO slim terms into biological process, cellular component and molecular function in zebrafish larvae exposed to 10 μM Arecoline (A), 10 μM Arecaidine (B), and 10 μM Arecoline *N*−oxide (C).

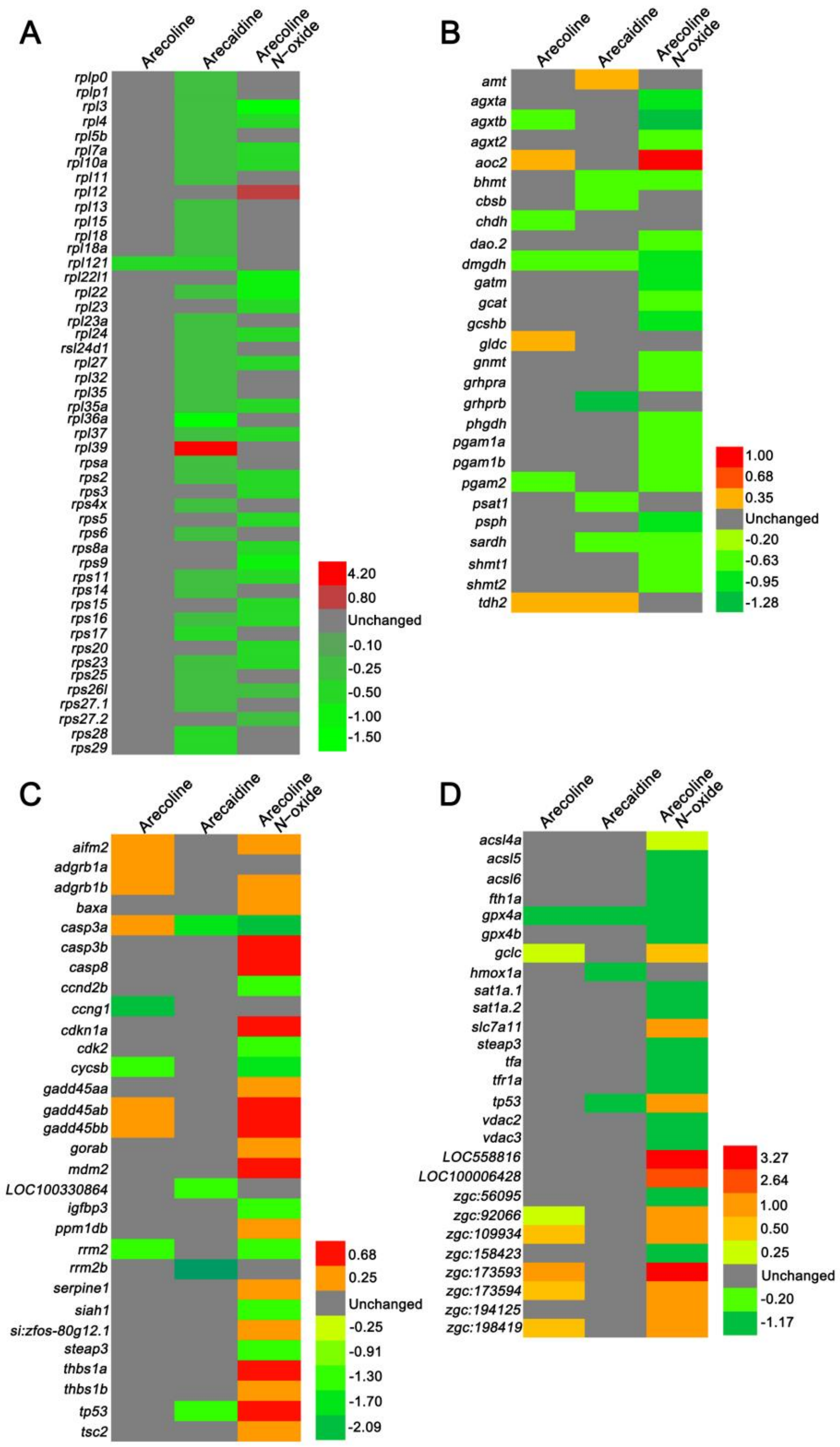

Figure S6. DEGs in the ribosome (A), glycine, serine and threonine metabolism (B), p53 signaling (C), and ferroptosis (D) pathways in zebrafish larvae after exposure to Areca−related toxins at 96 hpf.

Table S1. Hatchability of zebrafish larvae after exposure to areca alkaloids at 96 hpf

Arecoline	Mean(%)	SD	P value	Arecaidine	Mean(%)	SD	P value
Control	95.43	1.51		Control	95.51	1.64	
100 nM	91.60	1.39	0.0617 (ns)	100 nM	94.91	2.43	0.9940 (ns)
1 μM	93.91	1.56	0.7654 (ns)	1 μM	92.30	1.20	0.1483 (ns)
10 μM	94.64	0.08	0.9782 (ns)	10 μM	93.05	1.46	0.3570 (ns)
100 μM	95.61	1.53	0.9998 (ns)	100 μM	93.22	1.43	0.4260 (ns)
1 mM	91.92	2.57	0.0964 (ns)	1 mM	92.12	2.50	0.1166 (ns)
Arecoline *N*−oxide	Mean(%)	SD	P value				
Control	95.36	1.49					
100 nM	96.16	1.63	0.9770 (ns)				
1 μM	94.26	2.82	0.9190 (ns)				
10 μM	94.29	2.86	0.9264 (ns)				
100 μM	93.48	1.79	0.6062 (ns)				
1 mM	95.29	1.61	0.9999 (ns)				

Table S2. Mortality of zebrafish larvae after exposure to areca alkaloids at 96 hpf

Arecoline	Mean(%)	SD	P value	Arecaidine	Mean(%)	SD	P value
Control	0.95	1.65		Control	3.33	1.44	
100 nM	0.83	1.44	0.9999 (ns)	100 nM	2.50	2.50	0.9789 (ns)
1 µM	4.17	1.44	0.1857 (ns)	1 µM	2.50	2.50	0.9789 (ns)
10 µM	5.12	2.32	0.0535 (ns)	10 µM	4.17	1.44	0.9789 (ns)
100 µM	5.00	2.50	0.0633 (ns)	100 µM	1.67	1.44	0.7473 (ns)
1 mM	4.52	1.49	0.1198 (ns)	1 mM	5.00	2.50	0.7473 (ns)
Arecoline *N*−oxide	Mean(%)	SD	P value				
Control	2.62	2.51					
100 nM	0.95	1.65	0.7473 (ns)				
1 µM	0.95	1.65	0.7473 (ns)				
10 µM	1.91	1.65	0.9893 (ns)				
100 µM	2.62	2.51	0.9999 (ns)				
1 mM	1.91	1.65	0.9893 (ns)				

Table S3. Malformation of zebrafish larvae after exposure to areca alkaloids at 96 hpf

Arecoline	Mean(%)	SD	P value	Arecaidine	Mean(%)	SD	P value
Control	2.76	0.17		Control	2.68	0.16	
100 nM	10.92	1.38	0.0034 (**)	100 nM	18.26	1.53	< 0.001 (***)
1 μM	24.36	1.72	< 0.001 (***)	1 μM	21.15	1.51	< 0.001 (***)
10 μM	31.25	1.38	< 0.001 (***)	10 μM	46.67	5.95	< 0.001 (***)
100 μM	57.88	1.11	< 0.001 (***)	100 μM	86.17	2.22	< 0.001 (***)
1 mM	91.91	2.57	< 0.001 (***)	1 mM	95.29	1.61	< 0.001 (***)
Arecoline *N*−oxide	Mean(%)	SD	P value				
Control	2.81	0.16					
100 nM	18.26	1.53	< 0.001 (***)				
1 μM	21.15	1.51	< 0.001 (***)				
10 μM	46.67	5.95	< 0.001 (***)				
100 μM	86.17	2.22	< 0.001 (***)				
1 mM	95.29	1.61	< 0.001 (***)				

Table S4. Body length of the zebrafish larvae after exposure to areca alkaloids at 96 hpf

Arecoline	Mean(μm)	SD	P value	Arecaidine	Mean(μm)	SD	P value
Control	3891.04	93.56		Control	3817.13	113.56	
100 nM	3488.57	230.02	0.0129 (*)	100 nM	3278.02	81.12	<0.001 (***)
1 μM	3260.78	160.78	<0.001 (***)	1 μM	3099.11	121.38	<0.001 (***)
10 μM	3349.04	120.10	<0.001 (***)	10 μM	2827.09	447.99	<0.001 (***)
100 μM	3347.23	86.89	<0.001 (***)	100 μM	2965.23	359.81	<0.001 (***)
1 mM	3285.85	101.72	<0.001 (***)	1 mM	3039.15	155.88	<0.001 (***)
Arecoline *N*−oxide	Mean(μm)	SD	P value				
Control	3726.66	56.26					
100 nM	3715.03	111.30	0.9999 (ns)				
1 μM	3554.11	331.75	0.5539 (ns)				
10 μM	3551.11	165.66	0.5378 (ns)				
100 μM	3241.90	65.03	0.0018 (**)				
1 mM	2948.71	518.58	<0.001 (***)				

Table S5. Somatic axis bending of the zebrafish larvae after exposure to areca alkaloids at 96 hpf

Arecoline	Mean(%)	SD	P value	Arecaidine	Mean(%)	SD	P value
Control	2.76	0.17		Control	2.68	0.15	
100 nM	6.73	1.50	> 0.05 (ns)	100 nM	12.72	6.42	< 0.01 (**)
1 µM	13.16	0.35	< 0.01 (**)	1 µM	12.17	1.43	< 0.01 (**)
10 µM	20.53	1.42	< 0.001 (***)	10 µM	14.78	1.42	< 0.001 (***)
100 µM	45.64	3.30	< 0.001 (***)	100 µM	28.45	2.60	< 0.001 (***)
1 mM	68.53	3.32	< 0.001 (***)	1 mM	37.71	0.82	< 0.001 (***)
Arecoline *N*−oxide	Mean(%)	SD	P value				
Control	2.81	0.16					
100 nM	9.61	1.58	> 0.05 (ns)				
1 µM	13.47	1.78	< 0.001 (***)				
10 µM	27.42	4.36	< 0.001 (***)				
100 µM	54.61	2.91	< 0.001 (***)				
1 mM	63.15	6.16	< 0.001 (***)				

Table S6. Pericardial edema of the zebrafish larvae after exposure to areca alkaloids at 96 hpf

Arecoline	Mean(%)	SD	P value	Arecaidine	Mean(%)	SD	P value
Control	2.76	0.17		Control	2.68	0.15	
100 nM	6.73	1.50	> 0.05 (ns)	100 nM	15.49	6.18	< 0.001 (***)
1 μM	11.31	1.60	< 0.01 (**)	1 μM	16.81	1.41	< 0.001 (***)
10 μM	17.85	1.44	< 0.001 (***)	10 μM	24.60	2.13	< 0.001 (***)
100 μM	42.08	1.53	< 0.001 (***)	100 μM	44.33	1.59	< 0.001 (***)
1 mM	59.46	2.35	< 0.001 (***)	1 mM	50.93	3.36	< 0.001 (***)
Arecoline *N*−oxide	Mean(%)	SD	P value				
Control	2.81	0.16					
100 nM	11.51	2.73	< 0.01 (**)				
1 μM	16.36	1.81	< 0.001 (***)				
10 μM	30.48	4.36	< 0.001 (***)				
100 μM	59.22	1.82	< 0.001 (***)				
1 mM	66.93	4.80	< 0.001 (***)				

Table S7. Swim bladder loss of the zebrafish larvae after exposure to areca alkaloids at 96 hpf

Arecoline	Mean(%)	SD	P value	Arecaidine	Mean(%)	SD	P value
Control	2.76	0.17		Control	2.68	0.15	
100 nM	10.92	1.38	< 0.05 (*)	100 nM	22.21	1.04	< 0.001 (***)
1 μM	19.14	1.67	< 0.001 (***)	1 μM	26.49	0,94	< 0.001 (***)
10 μM	27.69	1.80	< 0.001 (***)	10 μM	33.90	2.18	< 0.001 (***)
100 μM	51.71	2.72	< 0.001 (***)	100 μM	59.25	7.26	< 0.001 (***)
1 mM	83.80	2.49	< 0.001 (***)	1 mM	81.64	4.09	< 0.001 (***)
Arecoline *N*−oxide	Mean(%)	SD	P value				
Control	2.81	0.16					
100 nM	15.38	1.54	< 0.001 (***)				
1 μM	18.26	1.53	< 0.001 (***)				
10 μM	39.05	4.36	< 0.001 (***)				
100 μM	75.11	4.53	< 0.001 (***)				
1 mM	85.85	2.87	< 0.001 (***)				

Table S8. Fold change in ROS in zebrafish larvae after exposure to areca alkaloids at 96 hpf (N=3)

Arecoline	Mean	SD	P value	Arecaidine	Mean	SD	P value
Control	1.000	0.057		Control	1.000	0.074	
1 μM	1.182	0.352	>0.05 (ns)	1 μM	1.177	0.187	>0.05 (ns)
10 μM	2.096	0.591	>0.05 (ns)	10 μM	2.568	0.583	< 0.01 (**)
100 μM	3.712	0.367	< 0.001 (***)	100 μM	5.798	0.561	< 0.001 (***)
1 mM	5.162	0.299	< 0.001 (***)	1 mM	7.856	0.562	< 0.001 (***)
Arecoline *N*−oxide	Mean	SD	P value				
Control	1.000	0.053					
1 μM	3.765	0.925	< 0.001 (***)				
10 μM	7.634	0.501	< 0.001 (***)				
100 μM	8.610	0.315	< 0.001 (***)				
1 mM	12.561	0.772	< 0.001 (***)				

Table S9. MDA of the zebrafish larvae after exposure to areca alkaloids at 96 hpf (N=4)

Arecoline	Mean(nmol/ mg weight)	SD	P value	Arecaidine	Mean(nmol/ mg weight)	SD	P value
Control	0.607	0.069		Control	0.582	0.021	
1 μM	0.672	0.089	>0.05 (ns)	1 μM	0.633	0.075	>0.05 (ns)
10 μM	0.821	0.053	< 0.001 (***)	10 μM	0.774	0.030	< 0.001 (***)
100 μM	1.025	0.127	< 0.001 (***)	100 μM	0.752	0.026	< 0.001 (***)
1 mM	1.287	0.053	< 0.001 (***)	1 mM	0.859	0.042	< 0.001 (***)
Arecoline *N*−oxide	Mean(nmol/ mg weight)	SD	P value				
Control	0.598	0.021					
1 μM	0.684	0.015	>0.05 (ns)				
10 μM	0.716	0.042	< 0.05 (*)				
100 μM	0.715	0.023	< 0.05 (*)				
1 mM	0.740	0.047	< 0.01 (**)				

Table S10. LPO of the zebrafish larvae after exposure to areca alkaloids at 96 hpf (N=4)

Arecoline	Mean(nmol/ mg weight)	SD	P value	Arecaidine	Mean(nmol/ mg weight)	SD	P value
Control	0.973	0.061		Control	0.825	0.061	
1 μM	1.441	0.035	< 0.001 (***)	1 μM	1.056	0.067	< 0.01 (**)
10 μM	2.036	0.078	< 0.001 (***)	10 μM	1.250	0.189	< 0.001 (***)
100 μM	2.590	0.152	< 0.001 (***)	100 μM	1.791	0.082	< 0.001 (***)
1 mM	3.016	0.053	< 0.001 (***)	1 mM	2.372	0.030	< 0.001 (***)
Arecoline *N*−oxide	Mean(nmol/ mg weight)	SD	P value				
Control	0.890	0.061					
1 μM	1.010	0.047	>0.05 (ns)				
10 μM	1.506	0.044	< 0.001 (***)				
100 μM	1.960	0.056	< 0.001 (***)				
1 mM	3.344	0.052	< 0.001 (***)				

Table S11. Fold change in GSH in zebrafish larvae after exposure to areca alkaloids at 96 hpf (N=4)

Arecoline	Mean	SD	P value	Arecaidine	Mean	SD	P value
Control	1.000	0.060		Control	1.000	0.035	
1 µM	0.898	0.035	< 0.05 (*)	1 µM	0.954	0.070	>0.05 (ns)
10 µM	0.797	0.048	< 0.001 (***)	10 µM	0.847	0.038	< 0.001 (***)
100 µM	0.570	0.025	< 0.001 (***)	100 µM	0.791	0.015	< 0.001 (***)
1 mM	0.470	0.091	< 0.001 (***)	1 mM	0.737	0.032	< 0.001 (***)
Arecoline *N*−oxide	Mean	SD	P value				
Control	1.000	0.034					
1 µM	0.991	0.031	>0.05 (ns)				
10 µM	0.739	0.050	< 0.001 (***)				
100 µM	0.631	0.048	< 0.001 (***)				
1 mM	0.602	0.040	< 0.001 (***)				

Table S12. Fold change in GSSG in zebrafish larvae after exposure to areca alkaloids at 96 hpf (N=4)

Arecoline	Mean	SD	P value	Arecaidine	Mean	SD	P value
Control	1.000	0.038		Control	1.000	0.031	
1 μM	0.945	0.041	>0.05 (ns)	1 μM	1.077	0.029	>0.05 (ns)
10 μM	1.088	0.044	< 0.05 (*)	10 μM	1.062	0.031	>0.05 (ns)
100 μM	1.085	0.051	< 0.05 (*)	100 μM	1.076	0.031	>0.05 (ns)
1 mM	1.130	0.077	< 0.001 (***)	1 mM	1.556	0.032	< 0.001 (***)
Arecoline *N*−oxide	Mean	SD	P value				
Control	1.000	0.028					
1 μM	1.081	0.029	< 0.05 (*)				
10 μM	0.946	0.009	>0.05 (ns)				
100 μM	1.027	0.033	>0.05 (ns)				
1 mM	1.247	0.047	< 0.001 (***)				

Table S13. GSH/GSSG of zebrafish larvae after exposure to areca alkaloids at 96 hpf (N=4)

Arecoline	Mean	SD	P value	Arecaidine	Mean	SD	P value
Control	0.973	0.061		Control	0.825	0.061	
1 μM	1.441	0.035	< 0.001 (***)	1 μM	1.056	0.067	< 0.01 (**)
10 μM	2.036	0.078	< 0.001 (***)	10 μM	1.250	0.189	< 0.001 (***)
100 μM	2.590	0.152	< 0.001 (***)	100 μM	1.791	0.082	< 0.001 (***)
1 mM	3.016	0.026	< 0.001 (***)	1 mM	2.372	0.030	< 0.001 (***)
Arecoline *N*−oxide	Mean	SD	P value				
Control	0890	0.061					
1 μM	1.010	0.047	< 0.05 (*)				
10 μM	1.506	0.044	< 0.001 (***)				
100 μM	1.960	0.056	< 0.001 (***)				
1 mM	3.344	0.052	< 0.001 (***)				

Table S14. Average movement distance per min of the zebrafish larvae in the light or dark after exposure to areca alkaloids at 96 hpf (N=15)

Light	Mean (mm)	SD	P value	Dark	Mean (mm)	SD	P value
Control	88.81	66.69		Control	167.2	46.92	
Arecoline	43.25	11.43	<0.001 (***)	Arecoline	66.83	17.76	<0.001 (***)
Arecaidine	63.21	14.11	<0.01 (**)	Arecaidine	130.8	16.99	<0.001 (***)
Arecoline N−oxide	52.31	11.66	<0.001 (***)	Arecoline N−oxide	63.25	17.67	<0.001 (***)

Table S15. Total movement distance of the zebrafish larvae after exposure to areca alkaloids at 96 hpf (N=15)

Group	Mean(mm)	SD	P value
Control	7679	795.2	
Arecoline	3302	291.1	<0.001 (***)
Arecaidine	5821	599.8	<0.001 (***)
Arecoline N−oxide	3457	412.6	<0.001 (***)

Table S16. Mean speed of the zebrafish larvae after exposure to areca alkaloids at 96 hpf (N=15)

Group	Mean(mm/min)	SD	P value
Control	128.0	13.25	
Arecoline	55.04	4.85	<0.001 (***)
Arecaidine	97.01	9.99	<0.001 (***)
Arecoline N−oxide	57.62	6.88	<0.001 (***)

Table S17. Primers of the selected genes for quantitative real−time PCR.

Gene name	Forward primer (5’−3’)	Reverse primer (5’−3’)
atf4	TGGATCTGGACTCTCTCCCG	ACTGGAACCTCAGTGTGCTC
atf6	GAGCCTCAGTCACCGTACTC	GCACACCTGGATGGGTCTTT
eif2s1a	ACCGCTTGGGTGTTTGATGA	CACCTCAATGTCTGCTCGGA
hspa5	ATGAAGACGTTTGCACCGGA	CTTTAGTGGCCTGACGCTGA
chop	CACAGACCCTGAATCAGAAG	CCACGTGTCTTTTATCTCCC
tp53	TGGAGATAACTTGGCGCCTG	ACCAAGCTGTGGTGCTTCAT
sqstm1	CCCTCCTGGTCCCTGTCATA	CAGGTGGGGCACAAGTCATA
bax	TGTATGAGCGTGTTCGTCGG	AGACGTCTTGAGTCGGCTG
bcl2	GCGGAGGGAACAACTCTGAA	CTCTTCGGCCACGGTTAGAA
caspase −3	GATCGCAGGACAGGCATGAA	CGTCATGGGCAACTGTTGTT
actb	CTCTGGTGATGGTGTGACCC	ATTTCTCTTTCGGCCGTGGT
